# Role of PPARα and HNF4α in Stress-Mediated Alterations in Lipid Homeostasis

**DOI:** 10.1371/journal.pone.0070675

**Published:** 2013-08-14

**Authors:** Maria Konstandi, Yatrik M. Shah, Tsutomu Matsubara, Frank J. Gonzalez

**Affiliations:** 1 Laboratory of Metabolism, National Cancer Institute, National Institutes of Health, Bethesda, Maryland, United States of America; 2 Department of Pharmacology, University of Ioannina, School of Medicine, Ioannina, Greece; 3 Department of Molecular and Integrative Physiology, University of Michigan, Ann Arbor, Michigan, United States of America; Baylor College of Medicine, United States of America

## Abstract

Stress is a risk factor for several cardiovascular pathologies. PPARα holds a fundamental role in control of lipid homeostasis by directly regulating genes involved in fatty acid transport and oxidation. Importantly, PPARα agonists are effective in raising HDL-cholesterol and lowering triglycerides, properties that reduce the risk for cardiovascular diseases. This study investigated the role of stress and adrenergic receptor (AR)-related pathways in PPARα and HNF4α regulation and signaling in mice following repeated restraint stress or treatment with AR-antagonists administered prior to stress to block AR-linked pathways. Repeated restraint stress up-regulated *Pparα* and its target genes in the liver, including *Acox*, *Acot1*, *Acot4*, *Cyp4a10*, *Cyp4a14 and Lipin2*, an effect that was highly correlated with *Hnf4α*. *In vitro* studies using primary hepatocyte cultures treated with epinephrine or AR-agonists confirmed that hepatic AR/cAMP/PKA/CREB- and JNK-linked pathways are involved in PPARα and HNF4α regulation. Notably, restraint stress, independent of PPARα, suppressed plasma triglyceride levels. This stress-induced effect could be attributed in part to hormone sensitive lipase activation in the white adipose tissue, which was not prevented by the increased levels of perilipin. Overall, this study identifies a mechanistic basis for the modification of lipid homeostasis following stress and potentially indicates novel roles for PPARα and HNF4α in stress-induced lipid metabolism.

## Introduction

The frequency of obesity related health risks, including hypertension, insulin resistance, type 2 diabetes, dyslipidemia, atherosclerosis, and cardiovascular disorders are increasing in Western societies [1]. Chronic stress is considered a risk factor for atherosclerosis, coronary artery disease and the entire spectrum of metabolic syndrome X, including visceral obesity, insulin resistance, dyslipidemia, dyscoagulation, and hypertension. The role of stress in the development of these pathologies is mainly attributed to disturbances in lipid and carbohydrate metabolism [2]–[9]. In particular, the chronic stress-induced hyperlipidemia, which is characterised by elevated plasma levels of cholesterol, low-density lipoproteins, triglycerides and low levels of high-density lipoprotein, has been connected with increased incidence of atherosclerosis, myocardial infarct and congestive heart failure [2], [3], [5], [10].

Circulating epinephrine and norepinephrine released from the adrenal medulla predominantly sets the tone of sympathetic system. Peripheral organs, receive sympathetic innervation with post-ganglionic neurons releasing norepinephrine [5]. These biogenic amines, along with glucocorticoids, major effectors of the stress system, inhibit glucose uptake, fatty acid storage, protein synthesis at storage sites, and stimulate the release of energy substrates, including glucose, amino acids and free fatty acids from muscle, adipose tissue and liver [2], [11], [12].

Peroxisome proliferator-activated receptors (PPARs), members of the nuclear receptor superfamily, directly regulate lipid, transport, storage and metabolism, glucose metabolism, adipogenesis, and inflammatory responses; they also modulate carcinogenesis [13], [14] Most notably, PPARs act as lipid sensors that translate changes in lipid/fatty acid levels into metabolic activity, leading to either fatty acid catabolism or lipid storage [15]–[17]. PPARs have been associated with obesity and type 2 diabetes [18]. In particular, PPARα, which is mainly expressed in the liver, kidney, and heart, is critical in lipid homeostasis by directly regulating genes involved in fatty acid uptake, ω-oxidation and β-oxidation [13], [18]. PPARα agonists, such as the fibrate class of drugs, are effective in the treatment of dyslipidemia, where they raise HDL and reduce serum triglycerides, properties that decrease incidence of atherosclerosis and reduce risk for the development of cardiovascular disorders [19]–[21]. It is worth noting that the FIELD trial [22] and ACCORD Lipid study [23] revealed that fibrates reduced nonfatal coronary events in patients at risk for cardiovascular disease, including those with type 2 diabetes. The significant benefit of the fenofibrate-simvastatin combination therapy over statins alone regarding the incidence of major cardiovascular events in patients with atherogenic ‘mixed’ dyslipidemia (moderate hypertriglyceridemia and low HDL-cholesterol) was especially important [23]. Moreover, there is accumulating evidence supporting a direct protective role for PPARα agonists in cardiomyocytes via the PPARα/IGF-1 pathway, an effect though, needing further investigation in humans [19]–[21], [24].

It was previously reported that exposure to acute restraint stress and glucocorticoids up-regulated PPARα in rats [25]. The present study investigated the role of repeated restraint stress and the major components of the stress system, glucocorticoids and adrenergic receptors on PPARα regulation. Emphasis was given on the role of adrenergic receptor-linked pathways, employing wild type and *Ppara*-null mice. The data showed that exposure to pscychophysiological stress modulated PPARα activity followed by alterations in serum lipid markers.

## Materials and Methods

### Animals

Adult male SV129 and *Ppara*-null mice [26], [27] were used in this study. Mice were fed NIH-31 rodent chow (Zeigler, Gardners, PA) ad libitum with continuous access to fresh drinking water and were housed up to five per cage under a standard 12-h light, 12-h dark cycle. Mice were monitored daily for outward signs of distress or adverse health effects. All animal studies were carried out in accordance with Institute of Laboratory Animal Resources (ILAR) guidelines and approved by the National Cancer Institute Animal Care and Use Committee.

### Restraint stress paradigm

Experimental animals were exposed to restraint stress two h daily (10:00–12:00) for four consecutive days in an isolated environment [28]. Throughout the stress task, animals were restricted to adequately ventilated individual 50 ml conical plastic tubes (3×10 cm Falcon tubes). They could rotate from supine to prone position but they could not turn head to tail. Non-stressed controls were left undisturbed, but food and water were removed for 2 h to match the drinking and feeding condition of the stress group.

### Drugs and treatment

Prazosin hydrochloride (Sigma-Aldrich, 20 mg/kg b.w., i.p.; Prazosin), an alpha_1_-AR blocker, or atipamezole hydrochloride (Antisedan, Orion Pharma, Pfizer, 200 µg/kg b.w., s.c.; Atipamezole), an alpha_2_-AR blocker were administered 30 min prior to the stress task. Propranolol hydrochloride (Sigma-Aldrich, 10 mg/kg, i.p.; propranolol), a beta-AR blocker, was administered 15 min before the stress. Immediately after the last stress task and 2 h after the last drug treatment, mice were killed by CO_2_ asphyxiation and trunk blood was collected in BD Microtainer Serum Separator Tubes (Becton, Dickinson and Company, USA) for hormonal and biochemical analyses. Liver and white adipose tissue samples were dissected for total RNA, total cellular protein, cytosolic and nuclear protein extraction. All tissue and serum samples were kept at −80°C until assayed. Hepatocyte α_1_-adrenergic receptor pathways were stimulated using the α_1_-AR agonist, phenylephrine hydrochloride (Sigma-Aldrich; PH). Isoprenaline hydrochloride (Sigma-Aldrich; ISOP) was used to activate beta-AR-linked pathways.

### Quantitative real-time PCR (qPCR)

Total RNA from livers and white adipose tissue was isolated using Trizol reagent (Invitrogen, Carlsbad, CA) following the manufacturer's protocol. The concentration of total RNA was determined spectrophotometrically. Quantitative real-time reverse transcriptase PCR (qPCR) was performed with cDNA generated from 1 µg total RNA with a SuperScript III reverse transcriptase kit (Invitrogen). Gene-specific primers were designed for qPCR using the Primer Express software (Applied Biosystems, Foster City, CA). The sequences for the forward and reverse primers used are shown in Table S1. SYBR Green PCR master mix (Applied Biosystems, Warrington, UK) was used for the real-time reactions, which were carried out using the ABI PRISM 7900 HT sequence detection system (Applied Biosystems). Relative mRNA expression levels were normalized to β-actin mRNA and absolute levels determined using the comparative threshold cycle method.

### Western blot analysis

Immunoblot analysis of PPARα and HNF4α was carried out using nuclear extracts of liver samples. For the preparation of the nuclear extracts, the NE-PER nuclear extraction kit (Pierce, Rockford, IL) was used. AKT and FOXO1 phosphorylation was assessed in nuclear and cytosolic proteins, while CREB and STAT5b phosphorylation was analyzed only in nuclear proteins and that of p-p70S6K was assessed only in cytosolic proteins. Protein concentrations were determined by BCA protein assay (Pierce, Rockford, IL). Proteins were subjected to sodium dodecyl sulfate-polyacrylamide gel electrophoresis and immunoblotting using the following antibodies: Rabbit monoclonal anti-mouse PPARα (Santa Cruz Biotechnology, Santa Cruz, CA), HNF4α goat polyclonal IgG (Santa Cruz), rabbit monoclonal phospho-AKT IgG (Ser473; Santa Cruz), rabbit polyclonal phospho-FOXO1 (Ser256; Santa Cruz Biotechnology), rabbit polyclonal phospho-CREB-1 IgG (Ser133; Santa Cruz Biotechnology), monoclonal anti-mouse phosphorylated STAT5b IgG (Tyr694, Cell Signaling Technology) and rabbit polyclonal p-p70S6K IgG (Thr 389; Cell Signaling Technology) were used. Immunoblotting with mouse *β*-actin, histone-H1 and -H3 antibodies (Santa Cruz Biotechnology) was used as loading control. As secondary antibodies, the anti-rabbit, anti-goat or anti-mouse IgG horseradish peroxidase conjugated antibodies (Cell Signaling Technology) were used and the proteins were detected using an enhanced chemiluminescence detection kit (Thermo Scientific-Pierce, Rockford, IL).

### Preparation of hepatocyte cultures

Hepatocytes were prepared using a modified method based on a previous report [29] In brief, parenchymal hepatocytes were isolated from mice weighing 20–25 g using in situ perfusion. The isolated hepatocytes were suspended in Williams' Medium E supplemented with L-glutamine, penicillin and streptomycin and were plated at a density of 0.80–1.0×10^6^ cells in 60 mm diameter collagen type I coated dish (BIOCOAT, Cell Environment, Becton Dickinson Labware, UK). The viability of isolated cells was checked with trypan blue dye exclusion and those with viability higher than 85% just before plating were used. Hepatocytes were cultured at 37°C for 24 h under an atmosphere of humidified 5% CO_2_ in order to allow the cells to adhere to the dish. Time and dose response experiments started 24 hours later. Primary hepatocyte cultures were treated with either corticosterone or the AR-agonists, epinephrine (EPIN), PH and ISOP, at different doses (1–100 µM for EPIN, PH and ISOP) and for a period of time raging from 4–36 hours.

### Hormonal and biochemical determinations

Serum corticosterone concentrations were measured using the Corticosterone EIA kit (ACE, Cayman Chemical Company, USA). The detection limit was about 40 pg/ml and the intra-assay coefficient of variation (CV) was 4.1%. Serum triglycerides were determined using the ThermoTrace kit (Melburne, Austarlia). Serum total cholesterol concentration was measured with the Cholesterol EIA kit (Wako Diagnostics, Richmond, VA). The levels of non-esterified fatty acids in serum were determined using the NEFA C, EIA kit (Wako Chemicals GmbH, Neuss, Germany). Alanine Aminotransferase (ALT) and aspartate aminotransferase (AST) serum levels were measured with the DiscretePak ALT and AST Reagents kits (Catachem Inc, Bridgeport, CT). Serum catecholamine levels, norepinephrine (NE) and EPIN, were determined using the IBL-International EIA kit (Cayman Chemicals, USA).

### Statistical analysis

The data are presented as the mean ± SE and were analysed using one-way analysis of variance (ANOVA) followed by multiple comparisons with Bonferonni's and Tuckey's list honest significant difference methods. The significance level for all analyses was set at probability of less than 0.05. Moreover, correlation statistical analysis was performed using the Pearson's coefficient correlations in order to investigate possible correlations between alterations in the relative hepatic PPARα mRNA expression and those observed in relative HNF4α mRNA levels.

## Results

### Up-regulating effect of stress on PPARa expression: Role of AR-linked pathways

**Figure 1:**
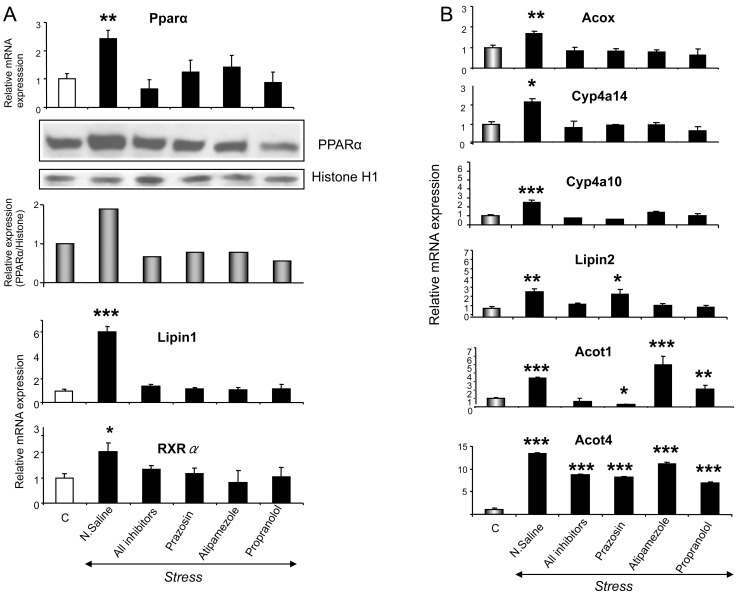
Stress-induced effect on *PPARα* and target gene expression. A. Following exposure of wild-type mice to restraint stress, *Ppara* mRNA levels were examined by qPCR analysis in their liver and PPARα protein was measured in the nuclear fraction by Western Blot analysis. Histone H1 served as a loading control. B. *Acox, Cyp4a10, Cyp4a14, Lipin2, Acot1*, *Acot4*, *Lipin1* and *RXRα* mRNA levels were also analyzed by qPCR in wild-type mice. Values were quantified using the comparative CT methods normalized to β-actin and are expressed as mean ± SE (n = 8–10). Comparisons were between controls and stress-exposed mice (alone or previously treated with the AR-antagonists, prazosin, propranolol or atipamezole). Group differences were calculated by one-way ANOVA, followed by Bonferonni's test. * P<0.025, **P<0.01, ***P<0.001.

Serum corticosterone and epinephrine levels were detected at higher levels in all stress-exposed animals compared to non-stressed controls (Table 1), indicating that all mice responded to stress [2]. The effect of stress on the expression of PPARα was determined by qPCR and western blot in the livers of wild-type mice. Restraint stress increased *Pparα* mRNA and PPARα protein levels (Fig. 1A) and this increase was prevented by the α_1_-AR antagonist, prazosin, the α_2_-AR antagonist, atipamezole and the beta-AR antagonist, propranolol (Fig. 1A). To test if the stress-induced *Pparα* expression could affect the expression of PPARα target genes, qPCR analysis of PPARα target gene mRNAs encoding enzymes critical in fatty acid homeostasis, were assessed. Stress markedly increased expression of hepatic acyl-coenzyme A oxidase (*Acox*), *Cyp4a10*, *Cyp4a14*, *Lipin 2*, cytosolic acyl-coenzyme A thioesterases *(Acot)1* and *Acot4* (Fig. 1B). Alpha- and beta-AR antagonists, given prior to stress, blocked the up-regulating effect of stress on *Acox, Cyp4a10* and *Cyp4a14* mRNAs (Fig. 1B). In the case of the stress-induced *Acot1* up-regulation, α_1_-ARs were found to have a major role as only prazosin blocked its induction (Fig. 1B). The stress-induced *Lipin2* up-regulation is mediated mainly by α_2_- and beta-ARs as atipamezole and propranolol blocked the up-regulating effect of stress (Fig. 1B). Since none of the AR-blockers used prevented the stress-induced *Acot4* up-regulation, it was likely not mediated by AR-related pathways, (Fig. 1B). Interestingly, exposure to restraint stress up-regulated in the liver the co-activator Lipin1 and retinoid X receptor alpha (RXRα) that forms heterodimers with PPARα. These effects were prevented by all AR-antagonists (Fig. 1B). In the liver of *Pparα* null mice, *Pparα*, *Cyp4a10* and *Acox* mRNA levels were detected at markedly lower levels compared to those detected in wild type mice and restraint stress did not affect them (P<0.01, Fig. S1).

**Table 1:** Role of PPARα in the stress-induced changes in serum lipid markers.

	Wild-type						*Ppara*-null	
	Control	Stress	Stress+ all AR-antagonists	Stress+ Prazosin	Stress+ Atipamezole	Stress+ Propranolol	Control	Stress
TG	141±13	83.2±8.0**	122±3.1	141±7.2	144±10	129±6.4	154±17	100±22
FFA	1.3±0.1	0.9±0.1**	1.5±0.3	1.8±0.4	1.4±0.2	1.3±0.1	2.0±0.3	1.9±0.3
T-Cholesterol	261±55	181±5.3**	265±78	194±20	255±46	305±14	314±61	291±6.5
Glucose	147±4.6	189±8.2**	151±10	101±11	154±11	157±17	155±5.7	192±7.8**
ALT	14.8±3.4	22.0±2.6	15.2±3.2	16.7±4.1	14.9±4.7	16.3±7.2	15.0±2.9	24.1±2.6
AST	46.8±7.2	62.0±9.5	57.3±13	64.3±8.9	48.8±15	54.2±14	56.5±8.6	79.4±14
CORT	68.4±7.3	110.4±11**	94.8±13*	115±28*	121±18**	95.6±17*	70.7±13	129±26*
EPIN	0.3±0.08	1.1±0.3***	0.2±0.01	0.2±0.01	0.2±0.0	0.2±0.01	0.4±0.1	1.4±0.2***
NE	2.9±0.8	3.3±0.4	2.5±0.3	2.5±0.2	2.6±0.1	2.4±0.3	3.1±0.9	3.7±1.2

Triglycerides (TG), free fatty acids (FFA) and total cholesterol (T-cholesterol). Alanine aminotransferase (ALT), aspartate aminotransferase (AST), corticosterone (CORT), epinephrine (EPIN), norepinephrine (NE), (WT, n = 20; *Ppara*-null, n = 12).

* P<0.05,

** P<0.01.

### Alterations in serum lipid indices indicative of PPARα activation

PPARα activation by stress resulted in suppression of plasma triglycerides (TG), free fatty acids (FFA) and total cholesterol levels, whereas blockade of ARs during stress, diminished the suppressive effect of stress on these lipids (Table 1). No significant changes in plasma FFA and total cholesterol concentrations were detected in *Ppara*-null mice following stress, again indicating a role for PPARα (Table 1). Nonetheless, it is of interest to note that the stress-mediated decrease in plasma TG levels was also detected in the plasma of *Ppara*-null mice (Table 1), indicating that PPARα is potentially not involved in this effect. No significant changes in serum AST, ALT, and body weights were observed following stress or drug treatment, thus revealing that the alterations observed in this study are not associated with any toxic effect or starvation (Table 1 and Table S2).

### *In vitro* assessment of hepatocyte adrenoceptor involvement in PPARα regulation

**Figure 2:**
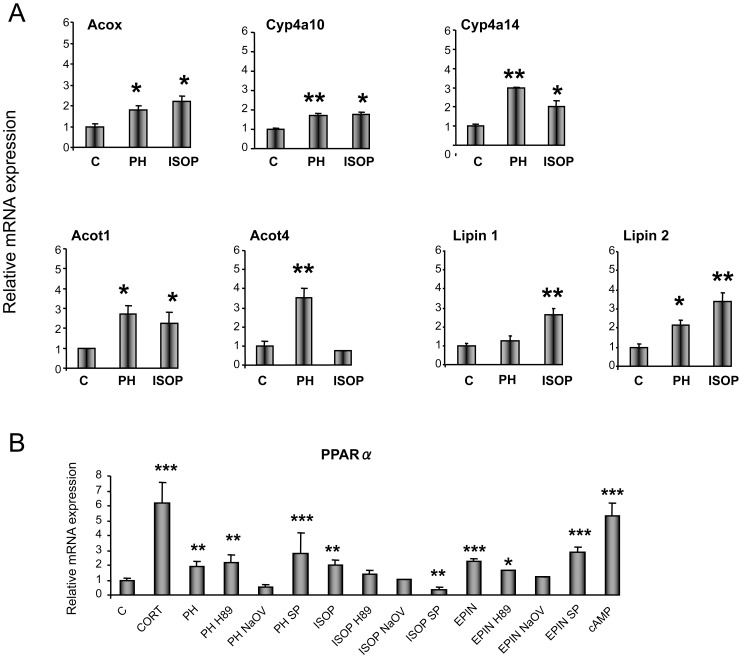
*In vitro* evaluation of the role of glucocorticoids and hepatic adrenergic receptor-linked pathways in *PPARα* and target gene expression. α. Following treatment of primary hepatocyte cultures with adrenergic receptor agonists for 24 hours, *Acox, Cyp4a10, Cyp4a14, Acot1, Acot4*, *Lipin1* and *Lipin2* mRNA levels were analyzed by qPCR. B. *Ppara* mRNA levels were also determined by qPCR in primary hepatocytes treated with either corticosterone, epinephrine or AR-agonists for 24 hours. Primary hepatocytes were also treated with AR-agonists in combination with the JNK inhibitor, SP600125 (SP), or the PKA inhibitors, H89 or sodium orthovanadate (NaOV). Values were quantified using the comparative CT methods normalized to *β*-actin and are expressed as mean ± SE (n = 3–4). Experiments were repeated three times. Comparisons were between control (DMSO) and drug-treated hepatocytes. C: control, CORT: corticosterone, PH: phenylephrine (alpha_1_-AR agonist), ISOP: isoprenaline (beta-AR agonist), EPIN: epinephrine (alpha- and beta-AR agonist), cAMP: 8-Br-cAMP. Group differences were calculated by one-way ANOVA, followed by Bonferonni's test. * P<0.025, **P<0.01, ***P<0.001.

To investigate whether the EPIN-stimulated hepatic PPARα up-regulation is due to a direct effect on hepatocyte ARs, primary hepatocytes were cultured in the presence of EPIN. The involvement of the specific AR-linked pathways in PPARα and HNF4α regulation was assessed by treating primary hepatocytes with the AR-agonists, PH and ISOP. Stimulation of alpha_1_- and beta-ARs, induced *Acox, Cyp4a10, Cyp4a14, Acot1 and Lipin2* mRNA levels in treated hepatocytes (Fig. 2A). *Acot4* mRNA was up-regulated by only PH, and *Lipin1* by only ISOP (Fig. 2A). Both AR-agonists used markedly induced *Ppara* expression in primary hepatocytes (Fig. 2B). Pretreatment of primary hepatocytes with the PKA inhibitor, sodium orthovanadate (NaOV, Sigma-Aldrich), prevented the PH-induced *Pparα* up-regulation (Fig. 2B). The ISOP-induced effect on *Ppara* expression was blocked by the JNK inhibitor, SP600125 (Enzo) and the PKA inhibitors, H89 (Sigma-Aldrich) and NaOV, (Fig. 2B). EPIN also markedly increased *Ppara* expression in hepatocytes and this effect was blocked by pretreatment with NaOV (Fig. 2B). The up-regulating effect of the cAMP/PKA pathway on *Pparα* expression was confirmed by treatment of primary hepatocytes with 8-Br-cAMP (Sigma-Aldrich) (Fig. 2B). Corticosterone also induced *Ppara* mRNA in primary hepatocyte cultures (Fig. 2B) in accordance with earlier studies [25].

### *In vivo* assessment of the stress-induced effect on several major signal transduction pathways related to lipid homeostasis and Pparα regulation

**Figure 3:**
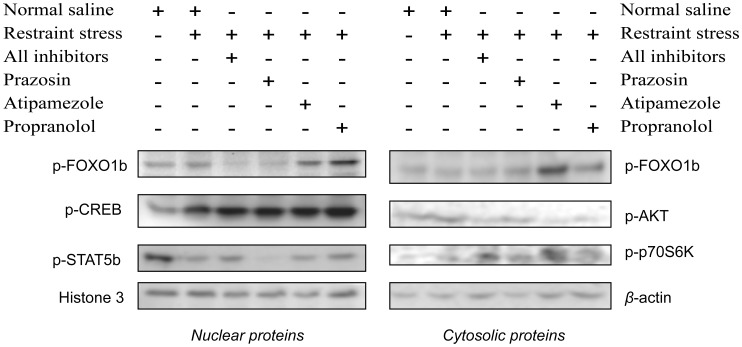
Stress-induced effect on PI3K/AKT, cAMP/PKA/CREB and GH/STAT5b signaling pathways. The evaluation of the stress effect on theses pathways was conducted in nuclear and cytosolic proteins with Western blot. Histone H3 served as a loading control for nuclear proteins and β-actin for cytosolic proteins. AR: adrenergic receptor; AR-antagonists given prior to stress inhibited α_1_-, α_2_- and beta-AR signaling; prazosin (α_1_-AR ainhibitor), atipamezole (α_2_-AR inhibitor), propranolol (β-AR inhibitor).

The insulin/PI3k/Akt signaling pathway has a critical role in the regulation of several genes encoding critical factors in TG synthesis [30], [31]. Exposure to restraint stress increased Akt phosphorylation in the liver (Fig. 3). However, this effect was not blocked by α_1_-AR inhibition, but only by α_2_- and beta-AR blockade (Fig. 3). Interestingly, the forkhead box protein O1 beta (FOXO1b) phosphorylation and that of p70S6K was not altered by stress, thus indicating that other downstream elements in the PI3k/Akt signaling pathway are involved in the stress-induced PPARα activation and synthesis of factors with essential roles in lipid homeostasis (Fig. 3). Interestingly, inhibition of α_2_-ARs prior to stress increased p70S6K activation, but this effect was not followed by any alteration in PPARα activation and lipid-related gene regulation (Fig. 3).

Stress also increased the cAMP response element-binding protein (CREB) phosphorylation in the liver (Fig. 3), although the increase was not prevented by the AR-inhibitors used in this study (Fig. 3). It is of interest also to note that restraint stress decreased the growth hormone (GH) pulse activated signal transducer and activator of transcription 5b (STAT5b) phosphorylation in the liver, which was not inhibited by the AR-antagonists used (Fig. 3).

### *In vivo* and *in vitro* assessment of the role of HNF4α in the stress-induced alterations in lipid homeostasis

**Figure 4:**
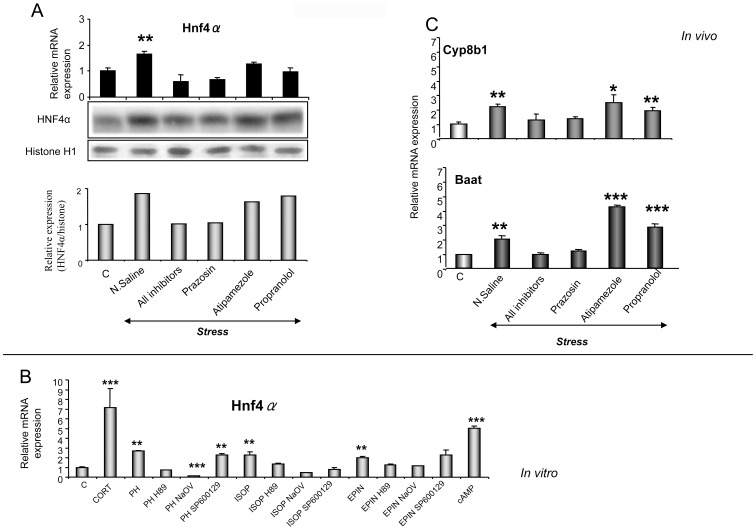
Stress-induced effect on *Hnf4a* expression. A. Following restraint stress alone or coupled with AR-antagonists hepatic *Hnf4a* mRNA levels were analysed in wild-type mice by qPCR. HNF4α protein was measured in liver nuclear fractions by Western blot analysis. Histone H1 served as a loading control. B. *Cyp8b1* and *Baat* mRNA levels were analyzed in the livers of wild-type mice by qPCR following treatment with either restraint stress alone or coupled with AR-antagonists (dark bars). C. *Hnf4a* mRNA levels were determined by qPCR following treatment of primary hepatocyte cultures with either corticosterone (CORT), epinephrine (EPIN) or AR-agonists for 24 hours. Primary hepatocytes were also treated with AR-agonists in combination with the JNK inhibitor, SP600125 (SP), or the PKA inhibitors, H89 or sodium orthovanadate (NaOV). Values were normalized to β-actin and are expressed as mean ± SE (n = 8–10). Comparisons were between controls and stress- or drug-treated mice. AR: adrenergic receptor, C: control, N. Saline: normal saline, All inhibitors: mice were treated with all AR-antagonists prior to stress, Prazosin (alpha_1_-AR antagonist), Atipamezole (alpha_2_-AR antagonist), Propranolol (beta-AR antagonist), PH: Phenylephrine (alpha_1_-AR agonist), ISOP: Isoprenaline (beta-AR agonist). Group differences were calculated by one-way ANOVA, followed by Bonferonni's test. *P<0.025, **P<0.01, ***P<0.001.

HNF4α and PPARα coordinate regulation of several common target genes [32]. In order to assess the involvement of HNF4α in the stress-induced up-regulation of PPARα target genes, hepatic RNA was analyzed by qPCR, and nuclear proteins quantified by Western blotting. HNF4α mRNA and protein levels were increased in the liver of restrained mice compared to non-stressed animals (Fig. 4A). The stress-induced up-regulation of *Hnf4α* was blocked only by the alpha_1_-AR antagonist, prazosin, whereas atipamezole and propranolol had no affect thus indicating a prevalent role for alpha_1_-AR-related pathways in the stress-induced *Hnf4α* up-regulation (Fig. 4A). The stress-induced *Hnf4a* expression triggered up-regulation of the HNF4α target genes, bile acid CoA (*Baat*) and *Cyp8b1* (Fig. 4B). Further investigation revealed that the stress-released EPIN up-regulated *Hnf4α* by directly stimulating hepatocyte alpha_1_- and beta-ARs, as treatment of primary hepatocytes with either EPIN, PH or ISOP markedly induced hepatocyte *Hnf4a* expression (Fig. 4C). The PH- and ISOP- induced up-regulating effect on *Hnf4a* mRNA was blocked by pre-treatment with the PKA inhibitors, H89 and NaOV (Fig. 4C). The JNK inhibitor, SP600125, also prevented the ISOP-induced *Hnf4a* up-regulation (Fig. 4C). The EPIN-induced effect on *Hnf4a* was blocked by pre-treatment of the hepatocytes with either H89 or NaOV (Fig. 4C). The involvement of the cAMP/PKA signaling pathway in the regulation of *Hnf4α* was also apparent when the hepatocytes were treated with 8-Br-cAMP (Fig. 4C). Interestingly, corticosterone markedly increased *Hnf4a* mRNA in primary hepatocytes (Fig. 4C), similar to what was noted with *Ppara* mRNA. Pearson's coefficient correlation revealed that the *in vivo* stress-induced alteration in *Ppara* expression is highly correlated to that observed in *Hnf4a* expression (Fig. S2). On the other hand, basal Hnf4α mRNA expression raged at equivalent levels in wild type and *Pparα* null mice, and exposure to restraint stress up-regulated *Hnf4α* in both animal models, indicating that this effect was not mediated by *Pparα* (Fig. S1 and Fig. 4A).

### *In vivo* assessment of the stress-mediated effects on lipid beta-oxidation, cholesterol synthesis and LDLr expression

**Figure 5:**
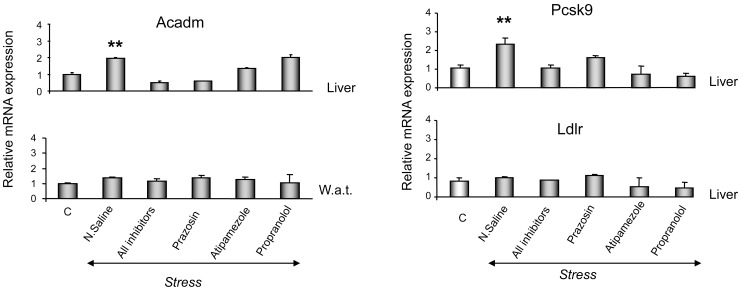
Stress-induced effect on *Acadm* expression. Effect of restraint stress and AR-related pathways on *Acadm* mRNA expression in the liver and in white adipose tissue (A), on Pcsk9 and Ldlr mRNA expression in the liver (B). Values were normalized to β-actin and are expressed as mean ± SE (n = 8–10). Comparisons were between controls and stress alone or coupled with AR-antagonists. AR: adrenergic receptor, C: control, N. Saline: normal saline, All inhibitors: mice were treated with all AR-antagonists prior to stress, prazosin (alpha_1_-AR antagonist), atipamezole (alpha_2_-AR antagonist), propranolol (beta-AR antagonist). **P<0.01, ***P<0.001.

Exposure to stress up-regulated acyl-coenzyme A dehydrogenase (*Acadm*) mRNA in the mouse liver (Fig. 5A) and this effect was completely blocked only by the alpha_1_-AR antagonist, prazosin (Fig. 5A). In contrast, stress did not alter *Acadm* expression in the white adipose tissue.

Stress also markedly increased the protein convertase subtilisin/kexin type 9 (Pcsk9) mRNA transcripts in the liver, an effect that was completely inhibited by all AR-antagonists used (Fig. 5B). In contrast, low-density lipoprotein receptor (Ldlr) expression was not affected by stress (Fig. 5B).

### *In vivo* assessment of the role of stress in TG homeostasis

**Figure 6:**
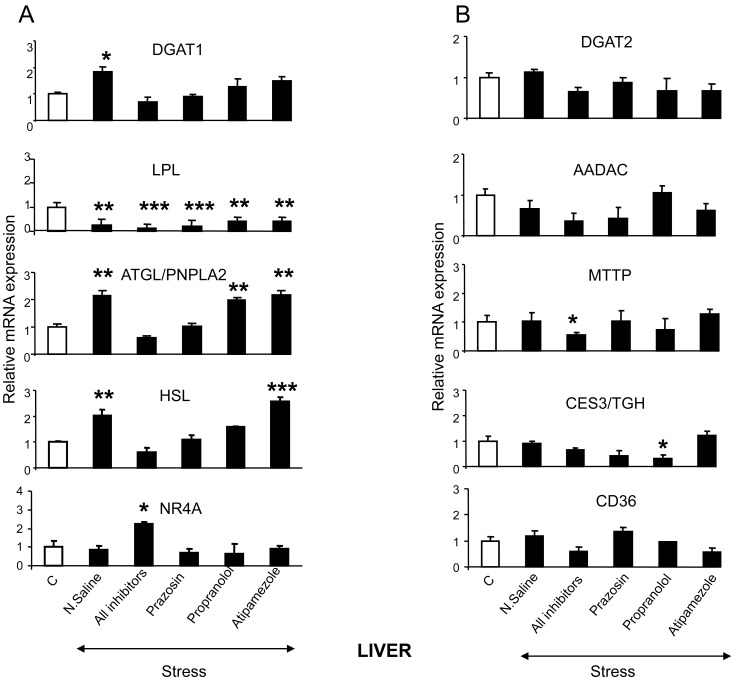
Stress-induced effect on hepatic TG homeostasis. (A) Effect of restraint stress on genes involved in TG synthesis and lipolysis in the liver. (B) Effect of restraint stress on genes involved in TG metabolism and clearance in the liver. Comparisons were between controls and stress-exposed mice (alone or simultaneously treated with the AR-antagonists, prazosin, propranolol or atipamezole; black bars). DGAT1: Diacyl glycerol acyltransferase 1 (acyl coenzyme A (CoA)), DGAT2: Diacyl glycerol acyltransferase 2, LPL: lipoprotein lipase, HSL: hormone sensitive lipase, ATGL/PNPLA2: adipose triglyceride lipase/patatin-like phospholipase domain containing 2, orphan nuclear receptor NR4A, AADAC: arylacetamide deacetylase, CD36: cluster of differentiation 36 or fatty acid transporter, CES3/TGH: carboxylesterase 3, MTTP: microsomal triglyceride transfer, PLIN5: perilipin 5. C: Control, N.Saline: normal saline, Prazosin (alpha_1_-AR antagonist), Atipamezole (alpha_2_-AR antagonist), Propranolol (beta-AR antagonist). Values are expressed as mean ± SE, n:5–6 per treatment group. *P<0.005, **P<0.01, ***P<0.001.

To further elucidate the mechanism underlying the suppressive effect of stress on TG serum concentration, the expression of various genes encoding factors involved in TG synthesis, metabolism and clearance were determined by qPCR and western blot analysis. The data revealed that stress increased *Dgat1*, *Atgl/Pnpla2* and *Hsl* mRNA expression in hepatic tissue (Fig. 6A). The stress-induced effect on *Dcat1* and *Hsl*, was blocked by prazosin and propranolol, and that on *Atgl/Pnpla2* was blocked only by prazosin (Fig. 6A). In contrast, stress suppressed *Lpl* mRNA levels in the liver (Fig. 6A).

**Figure 7:**
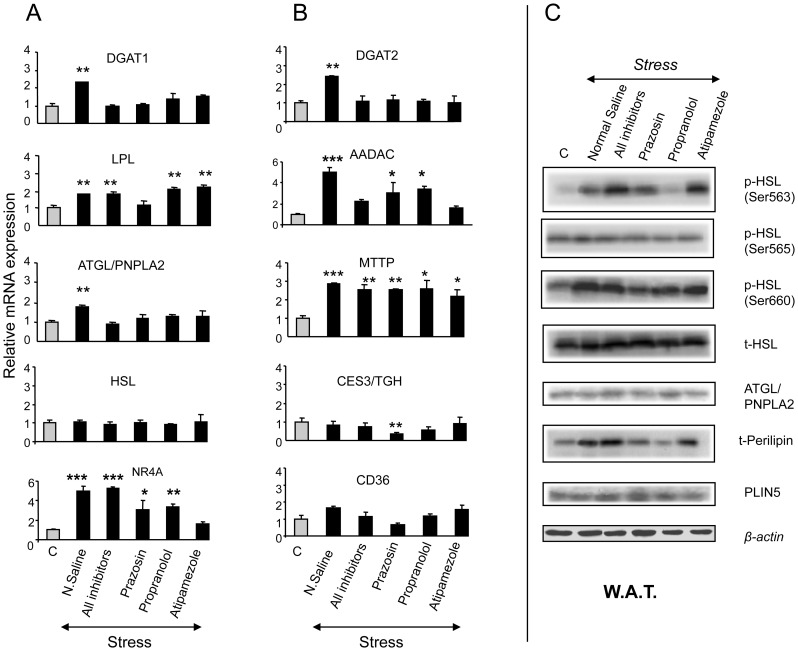
Stress-induced effect on TG homeostasis-related gene expression in the white adipose tissue. (A) Effect of restraint stress on the expression of genes involved in TG synthesis and lipolysis. (B) Effect of restraint stress on the expression of genes involved in TG metabolism and clearance. (C) Effect of restraint stress on the expression and activation of genes involved in TG hydrolysis. Comparisons were between controls and stress-exposed mice (alone or treated simultaneously with the AR-antagonists, prazosin, propranolol or atipamezole; black bars). DGAT1: Diacyl glycerol acyltransferase 1 (acyl coenzyme A (CoA), DGAT2: Diacyl glycerol acyltransferase 2, LPL: lipoprotein lipase, HSL: hormone sensitive lipase, ATGL/PNPLA2: adipose triglyceride lipase/patatin-like phospholipase domain containing 2, orphan nuclear receptor NR4A, AADAC: arylacetamide deacetylase, CD36: cluster of differentiation 36 or fatty acid transporter, CES3/TGH: carboxylesterase 3, MTTP: microsomal triglyceride transfer, PLIN5: perilipin 5. AR: adrenergic receptor, C: Control, Prazosin (alpha_1_-AR antagonist), Atipamezole (alpha_2_-AR antagonist), Propranolol (beta-AR antagonist), white adipose tissue (W.A.T.). Values are expressed as mean ± SE, n:5–6 per treatment group. Lanes in western blots correspond to one sample per treatment and represent one sample of three separate samples tested in different blots. *P<0.005, **P<0.01, ***P<0.001.

It should be noted that compared to non-stressed controls, stress increased the *Dgat1*, *Lpl*, *Atgl/Pnpla2*, *Nr4a*, *Dgat2*, *Aadac* and *Mttp* mRNA transcripts in the white adipose tissue (Fig. 7A and 7B). Further investigation revealed that stress increased the HSL phosphorylation at Ser563 and Ser660, as well as Perilipin apoprotein levels in the white adipose tissue (Fig. 7C), clearly confirming the involvement of stress in TG homeostasis. The involvement of AR-related pathways in the stress-mediated alterations in TG homeostasis, was also investigated in animals treated with AR-antagonists prior to their exposure to restraint stress. The data revealed that alpha_1_-AR blockade by prazosin prevented the stress-induced *Dgat1*, *Lpl*, *Atgl/Pnpla2*, *Dgat2* and *Aadac* up-regulation in the white adipose tissue (Fig. 7A and 7B). Both, alpha_2_- and beta-AR-antagonists, atipamezole and propranolol, respectively, prevented the *Atgl/Pnpla2*, *Dgat2* and *Aadac* up-regulation in the white adipose tissue (Fig. 7A and 7B). In the case of the stress-induced *Nr4a* up-regulation in the white adipose tissue the involvement of alpha_2_-AR-related pathways appears to be prevalent (Fig. 7A). The stress-induced HSL phosphorylation at Ser563 was blocked by propranolol (Fig. 7C), whereas that at Ser660 was blocked by prazosin (Fig. 7C). Notably, both prazosin and propranolol, prevented the stress-induced increase in perilipin apoprotein levels in the white adipose tissue (Fig. 7C).

## Discussion

Genomic and biochemical data in the present study confirmed that stress modifies lipid homeostasis [2]–[10]. Serum lipid markers, such as free fatty acids and total cholesterol, were detected at lower concentrations in stress-exposed wild-type mice compared to non-stressed controls, whereas, no significant change was observed in *Pparα*-null mice. These observations clearly indicate a significant role for PPARα in the stress-induced alterations in lipid homeostasis, further elucidating the regulatory mechanisms involved in the maintenance of cellular lipid balance under normal physiological conditions, psychological stress, or in the context of disease states. The role of PPARα as a cellular “lipostat”, transducing changes in cellular lipid levels to the transcriptional regulation of target genes involved in fatty acid utilization [16], [33] was confirmed by the present study.

A previous investigation on the role of stress in *PPARα* regulation, revealed that glucocorticoids can induce expression of *Ppara* [25]. The present data confirmed the up-regulating effect of glucocorticoids on *Ppara* and further indicated the determinant role of EPIN in the stress-induced effect. Nonetheless, the role of the AR-linked pathways and, in particular, the role of alpha-ARs in the stress-induced regulation of PPARα signal transduction was not clear. Therefore, within the scope of the present study was investigation of the role of AR-related pathways, major components of the sympathoadrenal response to stress and targets of drugs used for the treatment of hypertension, asthma, angina pectoris, congestive heart failure, cardiac arrhythmias, prostatic hypertrophy, glaucoma, and depression [34], [35]. In this context, it is also of interest to note that ARs hold major physiological roles, such as the regulation of carbohydrate, lipid and amino acid metabolism in the liver, among others, but the mechanisms remain still unclear [36]–[39].

*In vivo* studies employing pharmacological manipulations of AR-related pathways using AR-antagonists given prior to stress, revealed a critical role for ARs in *Pparα* regulation by stress. In particular, blockade of alpha_1_-, alpha_2_- or beta-ARs during the stress manipulation, prevented the stress-induced effect on *Ppara* and PPARα target gene expression. The up-regulating effect of stress on PPARα and its target genes is potentially, in part, associated with the up-regulation of RXRα, which forms heterodimmers with PPARα. This complex binds to peroxisome proliferator response element (PPRE) in the promoter of target genes and stimulates their transcription [40]. Stress also induced *Lipin1* expression, which functions as a nuclear transcriptional coactivator with peroxisome proliferator-activated receptor α thus modulating the expression of several genes involved in lipid metabolism [41].

At the signal transduction level, the insulin/PI3k/Akt signalling pathway appears to have a role in the stress-induced regulation of PPARα and genes encoding factors involved in lipid homeostasis [30], [31]. Stress activated Akt in the liver via pathways not related to α_1_-ARs but to α_2_- and beta-ARs, as only atipamezole and propranolol blocked the stimulating effect of stress on Akt phosphorylation. Interestingly, activation of Akt appears to stimulate downstream elements in the PI3k/Akt pathway, other than FOXO1b and p70S6K, which mediate the up-regulating effects of stress.

*In vitro* stimulation of hepatocyte alpha_1_-AR/cAMP/PKA signaling pathway with PH, the beta-AR/cAMP/PKA and beta-AR/JNK-linked pathways with ISOP, was followed by up-regulation of PPARα and its target genes. To our knowledge, this is the report of a role for alpha_1_-ARs in the regulation of PPARα. *In vivo*, the stress-induced release of EPIN activated hepatic α_1_- and beta-AR/cAMP/PKA/CREB signaling pathway thus activating PPARα. It should be noted though that the AR-antagonists at the doses given, did not prevent the stress-induced effect. Potentially, other central and peripheral AR-linked pathways have overridden the effect of hepatic AR-linked pathways.

Stress also decreased phosphorylation of the GH-pulse activated transcription factor, STAT5b, in the liver, an effect though that was not blocked by the AR-inhibitors used, indicating that the stress-induced inactivation of STAT5b is mediated by glucocorticoids [42] and not by central or peripheral AR-linked pathways. The suppressive effect of stress on the GH/STAT5b pathway potentially mediates the stress-induced *Pparα* up-regulation. The bidirectional inhibitory cross-talk between PPAR and STAT5b is well documented [43].

The stress-induced *Pparα* and target gene up-regulation was followed by reduced plasma T-cholesterol, TG and FFA levels. The present data are consistent with previous reports indicating that stress reduced plasma TG levels [44]–[47]. The effect of stress on the above mentioned lipid markers appears to be mediated mainly by stimulation of AR-linked pathways with epinephrine as it was completely blocked by AR-antagonists. It is of interest to note that stress also suppressed plasma TG levels in *Ppara*-null mice without affecting plasma FFA and T-cholesterol levels, thus indicating that the stress-induced suppressive effect on TG is not exclusively mediated by PPARα. Several other transcription factors can contribute to the TG lowering effects of stress, including SREBP-1c, NF-kappaB, RXRs, LXRs, FXR and HNF4α [48]. The mechanism mediating the stress- and AR-related effect on lipid homeostasis remains unclear and currently a subject of thorough investigation. TG are stored in lipid droplets mainly in adipocytes, and during lipolysis they are hydrolized into free fatty acids and glycerol, a sequential process involving different lipases, mainly the HSL and ATGL, which accounts for most of the HSL-independent TG hydrolase activity in white adipose tissue [49]–[51]. In this process, perilipin holds a key role; it is localized at the periphery of lipid droplets serving as a protective coating against lipases [49], [50], [52], [53]. It was reported that stimulation of the cAMP/PKA signalling pathway may activate perilipin, thus leading to conformational alterations, which facilitate the exposure of lipid droplets to endogenous lipases [52].

In the cascade of events involved in the stress-induced significant decline in plasma TG levels, increased mitochondrial lipid beta-oxidation may be partly involved, as stress up-regulated *Acadm*, an enzyme that catalyzes fatty acid beta-oxidation [54], [55]. It is of interest to note that repeated restraint stress also induced expression of the nuclear receptor *Hnf4α*, which is central to the maintenance of hepatocyte differentiation and the regulation of genes involved in lipid metabolism [56], [57]. Importantly, as in the case of *Pparα*, the hepatocyte alpha_1_- and beta-AR/cAMP/PKA signaling pathway along with that of beta-AR/JNK-linked pathway appear to mediate *Hnf4α* up-regulation [58]. The *in vivo* and *in vitro* data indicated that the alpha_1_- and beta_1/2_-AR-induced *Hnf4α* up-regulation, should be attributed to a direct stimulation of hepatic alpha_1_- or beta_1/2_-AR signaling pathways by EPIN. The critical role of the hepatic ARs in *Hnf4α* regulation by stress is strengthened by the fact that stimulation of hypothalamic α_1_- or beta-ARs leads to suppressed plasma GH levels [59], a hormonal state connected with down-regulation of HNF4α target genes. A bidirectional cross-talk between the GH/STAT5b signalling and HNF4α is well documented. In particular, STAT5b activation has been connected with augmentation of HNF4α-dependent gene transcription, while HNF4α inhibits STAT5b transcriptional activity via inhibition of JAK2 phosphorylation [60]. In contrast, α_2_-ARs, expressed on pancreatic beta-cells, possess a positive control on insulin secretion [61], which in turn, via activation of hepatic mTOR signaling, has a negative control on HNF4α [62]. In addition, cytokines, which are released from immune cells following stimulation of all types of ARs have a suppressive effect on *Hnf4α* regulation [63]–[65]. Based on these reports and the present findings, it is apparent that the hepatic alpha_1_- and beta-AR-related effects on *Hnf4α* regulation override that of central and other peripheral AR-mediated effects. The findings of this study confirm the positive feedback regulatory mechanism involving PPARα and HNF4α that is activated when there is an increased demand for these two transcription factors to activate target genes [57].

Current evidence suggests that stress up-regulated several genes in the white adipose tissue, which are critical in the synthesis and metabolism of TG, such as the *Dgat1*, *Dgat2*, *Lpl*, *Atgl*, *Aadac*, *Mttp* and *Nr4a*. The stress-induced effect on *Lpl*, which has dual functions of TG hydrolase and ligand/bridging factor of receptor mediated lipoprotein up-take [52], [53], is apparently mediated via alpha_1_-ARs. Interestingly, the stress-induced up-regulating effect on *Dgat1*, *Dgat2*, *Aadac* and *Atgl* is mediated by alpha- and beta-ARs. It should be also noted that stress induced the *Nr4a* expression only via α_2_-ARs. Importantly, the stress activated HSL, one of the major enzymes contributing to TG breakdown in the white adipose tissue [66] and this effect was mediated by α_1_-and beta-ARs. Apparently, the stress-induced increase in perilipin concentration in the white adipose tissue, which was mediated by α_1_- and beta-AR-related pathways, did not prevent the effect of lipases and hydrolases on TG. Notably stress markedly up-regulated *Pcsk9* that plays a major role in cholesterol synthesis, but this increase apparently was not strong enough to affect LDLr and in turn, plasma cholesterol levels [62].

Taken together these studies revealed that short-term mild stress may be beneficial in lipid homeostasis, as it can stimulate an array of genes involved in lipid metabolism thus resulting in suppression of total cholesterol, triglycerides and free fatty acid plasma levels. This effect could be attributed to the overall adaptive mechanisms that are triggered by mild stress in order to enable the organism to maintain homeostasis [67]. In support of this notion are the findings of several previous studies indicating the beneficial impact of short-term mild stress in various disease states including cancer, immune diseases [68], Alzheimer's disease [69] and in situations that trigger oxidative damage [70]. Nonetheless, the long term detrimental effects of chronic stress should not be underestimated as it has long been considered as a crucial causative factor in the development of a myriad of diseases including those related to lipid and carbohydrate metabolism, with cardiovascular disorders and obesity holding significant portions of the stress-induced morbidity [2], [3], [5], [10]. From a clinical point of view, the challenge is the management of stress and not to let the sympathetic nervous system stay chronically aroused. A critical role for beta-ARs in PPARα activation was also well defined by previous [71] and the present data, which revealed a role for alpha_1_-ARs in *Pparα* regulation. These findings could be the basis for the design of new molecules targeting these receptors for PPARα activation. It should be also underscored the alpha- and beta-AR mediated stress-induced suppression in plasma TG levels, an effect that could be mainly attributed to increased TG lipolysis, metabolism and clearance. It is conceivable though that a thorough investigation of the mechanisms involved should be the centre of a new study. Overall, this study sheds more light on the complex pathophysiological states related to the stress-induced lipid disturbances and potentially suggests innovative therapeutic approaches indicating new targets in the regulation of *PPARα* and lipid homeostasis.

## Supporting Information

Table S1 **List of 5′ to 3′ oligonucleotide sequences used as forward and reverse primers.**
(DOC)

Table S2 **Alterations in the body weight after stress.** Body weight values are expressed in g.
(DOC)

Figure S1 **Stress-induced effect on** ***PPARα*** **expression in** ***Pparα*** **null mice.** A. PPARα mRNA and protein levels were examined in the liver of *Pparα* null mice followed restraint stress or treatment with AR agonists. B. Hepatic *Acox* and *Cyp4a10* mRNA levels were also analyzed by qPCR in these mice. C. HNF4α mRNA levels were examined in the liver of *Ppara*-null mice followed restraint stress or treatment with AR agonists. C: controls, PH: phenylephrine (α_1_-agonist), DEXT: dexmedetomidine (α_2_-agonist), ISOP: isoprenaline (β-agonist). Values were quantified using the comparative CT methods normalized to β-actin and are expressed as mean ± SE (n = 8–10). Comparisons took place between controls and stress-exposed or drug-treated mice. Group differences were calculated by one-way ANOVA, followed by Bonferonni's test. * P<0.025, **P<0.01.
(DOC)

Figure S2 **Correlation between stress- and AR-induced alterations in *PPARα* and *HNF4α* expression.** A. Correlation between stress-induced alterations in PPARα and HNF4α relative mRNA expression. B. Correlation between α_1_-AR-induced alterations in PPARα and HNF4α relative mRNA expression. C. Correlation between β-AR induced alterations in PPARα and HNF4α relative mRNA expression. D. Correlation of α_2_-AR induced alterations in PPARα and HNF4α mRNA relative expression.
(DOC)

## References

[1] Wang M (2005) The role of glucocorticoid action in the pathophysiology of the Metabolic Syndrome. Nutr Metab (Lond) 2: 3. 15689240 10.1186/1743-7075-2-3 PMC548667

[2] Johnson EO, Kamilaris TC, Chrousos GP, Gold PW (1992) Mechanisms of stress: a dynamic overview of hormonal and behavioral homeostasis. Neurosci Biobehav Rev 16: 115–130. 1630726 10.1016/s0149-7634(05)80175-7

[3] Tsigos C, Chrousos GP (1996) Differential diagnosis and management of Cushing's syndrome. Annual Review of Medicine 47: 443–461. 10.1146/annurev.med.47.1.443 8712794

[4] Friedman TC, Mastorakos G, Newman TD, Mullen NM, Horton EG, et al (1996) Carbohydrate and lipid metabolism in endogenous hypercortisolism: Shared features with metabolic syndrome X and NIDDM. Endocrine Journal 43: 645–655. 9075604 10.1507/endocrj.43.645

[5] Chrousos GP, Gold PW (1998) A healthy body in a healthy mind - and vice versa - The damaging power of “uncontrollable” stress. Journal of Clinical Endocrinology & Metabolism 83: 1842–1845. 9626106 10.1210/jcem.83.6.4908

[6] Rosmond R, Dallman MF, Bjorntorp P (1998) Stress-related cortisol secretion in men: Relationships with abdominal obesity and endocrine, metabolic and hemodynamic abnormalities. Journal of Clinical Endocrinology & Metabolism 83: 1853–1859. 9626108 10.1210/jcem.83.6.4843

[7] Karavanaki K, Tsoka E, Liacopoulou M, Karayianni C, Petrou V, et al (2008) Psychological stress as a factor potentially contributing to the pathogenesis of Type 1 diabetes mellitus. J Endocrinol Invest 31: 406–415. 18560258 10.1007/BF03346384

[8] Golden SH (2007) A review of the evidence for a neuroendocrine link between stress, depression and diabetes mellitus. Curr Diabetes Rev 3: 252–259. 18220683 10.2174/157339907782330021

[9] Ware WR (2008) High cholesterol and coronary heart disease in younger men: the potential role of stress induced exaggerated blood pressure response. Med Hypotheses 70: 543–547. 17714881 10.1016/j.mehy.2007.06.031

[10] Koch FS, Sepa A, Ludvigsson J (2008) Psychological stress and obesity. J Pediatr 153: 839–844. 18657829 10.1016/j.jpeds.2008.06.016

[11] Yates JC, Taam GML, Singal PK, Beamish RE, Dhalla NS (1980) Protection against Adrenochrome-Induced Myocardial Damage by Various Pharmacological Interventions. British Journal of Experimental Pathology 61: 242–255. 7426380 PMC2041585

[12] Guyre PM, Bodwell JE, Munck A (1984) Glucocorticoid actions on lymphoid tissue and the immune system: physiologic and therapeutic implications. Prog Clin Biol Res 142: 181–194. 6424133

[13] Yumuk VD (2006) Targeting components of the stress system as potential therapies for the metabolic syndrome - The peroxisome-proliferator-activated receptors. Stress, Obesity, and Metabolic Syndrome 1083: 306–318. 10.1196/annals.1367.019 17148746

[14] Shah YM, Morimura K, Yang Q, Tanabe T, Takagi M, et al (2007) Peroxisome proliferator-activated receptor alpha regulates a microRNA-mediated signaling cascade responsible for hepatocellular proliferation. Mol Cell Biol 27: 4238–4247. 17438130 10.1128/MCB.00317-07 PMC1900062

[15] Desvergne B, Michalik L, Wahli W (2004) Be fit or be sick: Peroxisome proliferator-activated receptors are down the road. Molecular Endocrinology 18: 1321–1332. 15087471 10.1210/me.2004-0088

[16] Michalik L, Auwerx J, Berger JP, Chatterjee VK, Glass CK, et al (2006) International Union of Pharmacology. LXI. Peroxisome proliferator-activated receptors. Pharmacological Reviews 58: 726–741. 17132851 10.1124/pr.58.4.5

[17] Evans RM, Barish GD, Wang YX (2004) PPARs and the complex journey to obesity. Nat Med 10: 355–361. 15057233 10.1038/nm1025

[18] Balint BL, Nagy L (2006) Selective modulators of PPAR activity as new therapeutic tools in metabolic diseases. Endocr Metab Immune Disord Drug Targets 6: 33–43. 16611163 10.2174/187153006776056620

[19] Robillard R, Fontaine C, Chinetti G, Fruchart JC, Staels B (2005) Fibrates. Handb Exp Pharmacol 389–406. 16596808 10.1007/3-540-27661-0_14

[20] Fruchart JC, Duriez P (2006) Mode of action of fibrates in the regulation of triglyceride and HDL-cholesterol metabolism. Drugs Today (Barc) 42: 39–64. 16511610 10.1358/dot.2006.42.1.963528

[21] Hansen MK, Connolly TM (2008) Nuclear receptors as drug targets in obesity, dyslipidemia and atherosclerosis. Current Opinion in Investigational Drugs 9: 247–255. 18311660

[22] Barter PJ, Rye KA (2008) Is there a role for fibrates in the management of dyslipidemia in the metabolic syndrome? Arteriosclerosis Thrombosis and Vascular Biology 28: 39–46. 10.1161/ATVBAHA.107.148817 17717290

[23] Saha SA, Arora RR (2011) Hyperlipidaemia and cardiovascular disease: do fibrates have a role? Current Opinion in Lipidology 22: 270–276. 21519250 10.1097/MOL.0b013e32834701c3

[24] Kuusisto J, Andrulionyte L, Laakso M (2007) Atherosclerosis and cardiovascular risk reduction with PPAR agonists. Curr Atheroscler Rep 9: 274–280. 18173954 10.1007/s11883-007-0033-4

[25] Lemberger T, Saladin R, Vazquez M, Assimacopoulos F, Staels B, et al (1996) Expression of the peroxisome proliferator-activated receptor alpha gene is stimulated by stress and follows a diurnal rhythm. Journal of Biological Chemistry 271: 1764–1769. 8576180 10.1074/jbc.271.3.1764

[26] Lee SS, Pineau T, Drago J, Lee EJ, Owens JW, et al (1995) Targeted disruption of the alpha isoform of the peroxisome proliferator-activated receptor gene in mice results in abolishment of the pleiotropic effects of peroxisome proliferators. Mol Cell Biol 15: 3012–3022. 7539101 10.1128/mcb.15.6.3012 PMC230532

[27] Akiyama TE, Nicol CJ, Fievet C, Staels B, Ward JM, et al (2001) Peroxisome proliferator-activated receptor-alpha regulates lipid homeostasis, but is not associated with obesity: studies with congenic mouse lines. J Biol Chem 276: 39088–39093. 11495927 10.1074/jbc.M107073200

[28] Konstandi M, Johnson EO, Marselos M, Kostakis D, Fotopoulos A, et al (2004) Stress-mediated modulation of B(alpha)P-induced hepatic CYP1A1: role of catecholamines. Chem Biol Interact 147: 65–77. 14726153 10.1016/j.cbi.2003.10.007

[29] Seglen PO (1976) Preparation of isolated rat liver cells. Methods Cell Biol 13: 29–83. 177845 10.1016/s0091-679x(08)61797-5

[30] Altomonte J, Cong L, Harbaran S, Richter A, Xu J, et al (2004) Foxo1 mediates insulin action on apoC-III and triglyceride metabolism. J Clin Invest 114: 1493–1503. 15546000 10.1172/JCI19992 PMC525736

[31] Cheng Z, White MF (2011) Targeting Forkhead box O1 from the concept to metabolic diseases: lessons from mouse models. Antioxid Redox Signal 14: 649–661. 20615072 10.1089/ars.2010.3370 PMC3025764

[32] Chamouton J, Latruffe N (2012) PPARalpha/HNF4alpha interplay on diversified responsive elements. Relevance in the regulation of liver peroxisomal fatty acid catabolism. Curr Drug Metab 13: 1436–1453. 22978398 10.2174/138920012803762738

[33] Rampler H, Weinhofer I, Netik A, Forss-Petter S, Brown PJ, et al (2003) Evaluation of the therapeutic potential of PPARalpha agonists for X-linked adrenoleukodystrophy. Mol Genet Metab 80: 398–407. 14654352 10.1016/j.ymgme.2003.09.002

[34] Virtanen R (1989) Pharmacological profiles of medetomidine and its antagonist, atipamezole. Acta Vet Scand Suppl 85: 29–37. 2571275

[35] Lalchandani SG, Zhang XY, Hong SS, Liggett SB, Li W, et al (2004) Medetomidine analogs as selective agonists for the human alpha(2)-adrenoceptors. Biochemical Pharmacology 67: 87–96. 14667931 10.1016/j.bcp.2003.08.043

[36] Cruise JL, Knechtle SJ, Bollinger RR, Kuhn C, Michalopoulos G (1987) Alpha-1-Adrenergic Effects and Liver-Regeneration. Hepatology 7: 1189–1194. 2824312 10.1002/hep.1840070604

[37] Minneman KP, Esbenshade TA (1994) Alpha(1)-Adrenergic Receptor Subtypes. Annual Review of Pharmacology and Toxicology 34: 117–133. 10.1146/annurev.pa.34.040194.001001 8042847

[38] Graham A, Angell ADR, Jepson CA, Yeaman SJ, Hassall DG (1996) Impaired mobilisation of cholesterol from stored cholesteryl esters in human (THP-1) macrophages. Atherosclerosis 120: 135–145. 8645354 10.1016/0021-9150(95)05695-5

[39] Luttrell LM, vanBiesen T, Hawes BE, Koch WJ, Krueger KM, et al (1997) G-protein-coupled receptors and their regulation - Activation of the MAP kinase signaling pathway by G-protein-coupled receptors. Signal Transduction in Health and Disease 31: 263–277. 9344257

[40] Chan LS, Wells RA (2009) Cross-Talk between PPARs and the Partners of RXR: A Molecular Perspective. PPAR Res 2009: 925309. 20052392 10.1155/2009/925309 PMC2801013

[41] Reue K (2009) The lipin family: mutations and metabolism. Curr Opin Lipidol 20: 165–170. 19369868 10.1097/MOL.0b013e32832adee5 PMC2875192

[42] Mazziotti G, Giustina A (2013) Glucocorticoids and the regulation of growth hormone secretion. Nat Rev Endocrinol 9: 265–276. 23381030 10.1038/nrendo.2013.5

[43] Shipley JM, Waxman DJ (2004) Simultaneous, bidirectional inhibitory crosstalk between PPAR and STAT5b. Toxicol Appl Pharmacol 199: 275–284. 15364543 10.1016/j.taap.2003.12.020

[44] Ricart-Jane D, Rodriguez-Sureda V, Benavides A, Peinado-Onsurbe J, Lopez-Tejero MD, et al (2002) Immobilization stress alters intermediate metabolism and circulating lipoproteins in the rat. Metabolism-Clinical and Experimental 51: 925–931. 12077743 10.1053/meta.2002.33353

[45] Hagopian K, Ramsey JJ, Weindruch R (2003) Caloric restriction increases gluconeogenic and transaminase enzyme activities in mouse liver. Experimental Gerontology 38: 267–278. 12581790 10.1016/s0531-5565(02)00202-4

[46] Lam TKT, Gutierrez-Juarez R, Pocai A, Bhanot S, Tso P, et al (2007) Brain glucose metabolism controls the hepatic secretion of triglyceride-rich lipoproteins. Nature Medicine 13: 171–180. 10.1038/nm1540 17273170

[47] Depke M, Fusch G, Domanska G, Geffers R, Voelker U, et al (2008) Hypermetabolic syndrome as a consequence of repeated psychological stress in mice. Endocrinology 149: 2714–2723. 18325986 10.1210/en.2008-0038

[48] Sanderson LM, de Groot PJ, Hooiveld GJ, Koppen A, Kalkhoven E, et al (2008) Effect of synthetic dietary triglycerides: a novel research paradigm for nutrigenomics. PLoS One 3: e1681. 18301758 10.1371/journal.pone.0001681 PMC2244803

[49] Greenberg AS, Egan JJ, Wek SA, Garty NB, Blanchettemackie EJ, et al (1991) Perilipin, a major hormonally regulated adipocyte-specific phosphoprotein associated with the periphery of lipid storage droplets. Journal of Biological Chemistry 266: 11341–11346. 2040638

[50] Zimmermann R, Lass A, Haemmerle G, Zechner R (2009) Fate of fat: The role of adipose triglyceride lipase in lipolysis. Biochimica Et Biophysica Acta-Molecular and Cell Biology of Lipids 1791: 494–500. 10.1016/j.bbalip.2008.10.005 19010445

[51] Gauthier MS, Miyoshi H, Souza SC, Cacicedo JM, Saha AK, et al (2008) AMP-activated protein kinase is activated as a consequence of lipolysis in the adipocyte - Potential mechanism and physiological relevance. Journal of Biological Chemistry 283: 16514–16524. 18390901 10.1074/jbc.M708177200 PMC2423258

[52] Brasaemle DL (2007) Thematic review series: adipocyte biology. The perilipin family of structural lipid droplet proteins: stabilization of lipid droplets and control of lipolysis. J Lipid Res 48: 2547–2559. 17878492 10.1194/jlr.R700014-JLR200

[53] Ducharme NA, Bickel PE (2008) Lipid droplets in lipogenesis and lipolysis. Endocrinology 149: 942–949. 18202123 10.1210/en.2007-1713

[54] Tolwani RJ, Farmer SC, Johnson KR, Davisson MT, Kurtz DM, et al (1996) Structure and chromosomal location of the mouse medium-chain acyl-CoA dehydrogenase-encoding gene and its promoter. Gene 170: 165–171. 8666240 10.1016/0378-1119(95)00882-9

[55] Wang YM, Zhang B, Xue Y, Li ZJ, Wang JF, et al (2010) The mechanism of dietary cholesterol effects on lipids metabolism in rats. Lipids Health Dis 9: 4. 20070910 10.1186/1476-511X-9-4 PMC2820024

[56] Dongol B, Shah Y, Kim I, Gonzalez FJ, Hunt MC (2007) The acyl-CoA thioesterase I is regulated by PPAR alpha and HNF4 alpha via a distal response element in the promoter. Journal of Lipid Research 48: 1781–1791. 17485727 10.1194/jlr.M700119-JLR200

[57] Martinez-Jimenez CP, Kyrmizi I, Cardot P, Gonzalez FJ, Talianidis I (2010) Hepatocyte nuclear factor 4alpha coordinates a transcription factor network regulating hepatic fatty acid metabolism. Mol Cell Biol 30: 565–577. 19933841 10.1128/MCB.00927-09 PMC2812226

[58] Sinal CJ, Tohkin M, Miyata M, Ward JM, Lambert G, et al (2000) Targeted disruption of the nuclear receptor FXR/BAR impairs bile acid and lipid homeostasis. Cell 102: 731–744. 11030617 10.1016/s0092-8674(00)00062-3

[59] Tuomisto J, Mannisto P (1985) Neurotransmitter regulation of anterior pituitary hormones. Pharmacological Reviews 37: 249–332. 2869509

[60] Park SH, Wiwi CA, Waxman DJ (2006) Signalling cross-talk between hepatocyte nuclear factor 4alpha and growth-hormone-activated STAT5b. Biochem J 397: 159–168. 16584384 10.1042/BJ20060332 PMC1479742

[61] Morrow LA, Rosen SG, Halter JB (1991) Beta-adrenergic regulation of insulin secretion: evidence of tissue heterogeneity of beta-adrenergic responsiveness in the elderly. J Gerontol 46: M108–113. 1649216 10.1093/geronj/46.4.m108

[62] Ai D, Chen C, Han S, Ganda A, Murphy AJ, et al (2012) Regulation of hepatic LDL receptors by mTORC1 and PCSK9 in mice. J Clin Invest 122: 1262–1270. 22426206 10.1172/JCI61919 PMC3314476

[63] Flierl MA, Rittirsch D, Nadeau BA, Chen AJ, Sarma JV, et al (2007) Phagocyte-derived catecholamines enhance acute inflammatory injury. Nature 449: 721–725. 17914358 10.1038/nature06185

[64] Flierl MA, Rittirsch D, Huber-Lang M, Sarma JV, Ward PA (2008) Catecholamines-crafty weapons in the inflammatory arsenal of immune/inflammatory cells or opening pandora's box? Mol Med 14: 195–204. 18079995 10.2119/2007-00105.Flierl PMC2136428

[65] Flierl MA, Rittirsch D, Nadeau BA, Sarma JV, Day DE, et al (2009) Upregulation of phagocyte-derived catecholamines augments the acute inflammatory response. PLoS One 4: e4414. 19212441 10.1371/journal.pone.0004414 PMC2636885

[66] Schweiger M, Schreiber R, Haemmerle G, Lass A, Fledelius C, et al (2006) Adipose triglyceride lipase and hormone-sensitive lipase are the major enzymes in adipose tissue triacylglycerol catabolism. J Biol Chem 281: 40236–40241. 17074755 10.1074/jbc.M608048200

[67] Le Bourg E (2010) It Is Time to Thoroughly Study the Effects of Mild Stress in Rodents, but Also in Human Beings. Dose-Response 8: 64–67. 10.2203/dose-response.09-042.LeBourg PMC2836157 20221291

[68] Dhabhar FS (2009) Enhancing versus suppressive effects of stress on immune function: implications for immunoprotection and immunopathology. Neuroimmunomodulation 16: 300–317. 19571591 10.1159/000216188 PMC2790771

[69] Pardon MC, Sarmad S, Rattray I, Bates TE, Scullion GA, et al (2009) Repeated novel cage exposure-induced improvement of early Alzheimer's-like cognitive and amyloid changes in TASTPM mice is unrelated to changes in brain endocannabinoids levels. Neurobiol Aging 30: 1099–1113. 18023506 10.1016/j.neurobiolaging.2007.10.002

[70] Aschbacher K, O'Donovan A, Wolkowitz OM, Dhabhar FS, Su Y, et al (2013) Good stress, bad stress and oxidative stress: Insights from anticipatory cortisol reactivity. Psychoneuroendocrinology 10.1016/j.psyneuen.2013.02.004 PMC4028159 23490070

[71] Tateishi K, Okada Y, Kallin EM, Zhang Y (2009) Role of Jhdm2a in regulating metabolic gene expression and obesity resistance. Nature 458: 757–761. 19194461 10.1038/nature07777 PMC4085783

