# Supplementary material for: Role of PPARα and HNF4α in Stress-Mediated Alterations in Lipid Homeostasis
Source: PLoS One. 2013 Aug 14;8(8):e70675. doi: 10.1371/journal.pone.0070675 (PMC3743822; doi:10.1371/journal.pone.0070675)
Supplement: Table S1 — List of 5′ to 3′ oligonucleotide sequences used as forward and reverse primers. (DOC) [file pone.0070675.s001.doc]

**Table S1**

List of 5΄ to 3΄ oligonucleotide sequences used as forward and reverse primers

PPARα CAGTGGGGAGAGAGGACAGA AGTTCGGGAACAAGACGTTG

ACOT1 ATGGCAGCAGCTCCAGACTT CCCAACCTCCAAACCATCAT

ACOT4 AACATCGATGATGCCTGGA GTCACTTCATGGCTCCCG

Cyp4a14 GGTGAGGCTGATTGAGTCTTGAG CTCCAGATTGATCCAGGATGGA

Cyp4a10 CCAGGAACTGCATTGGGAAA GACCCTGGTAGGATCTGGCA

ACOX GGGAGTGCTACGGGTTACATG CCGATATCCCCAACAGTGATG

HNF4α CGGAGCCCCTGCAAAGT ACTATCCAGTCTCACAGCCCATTC

Cyp8b1 ACGCTTCCTCTATCGCCTGAA GTG CCTCAGACGCAGAGGAT

BAAT ACAGGCCTGGCCCCCTTTCA CCCATGGGGTGGACCCCCAT

RXRα CAGTACGCAAAGACCTGACCTACA GTTCCGCTGTCTCTTGTCGAT

Lipin1 TCCTTCACCGTCACAAACAC TTTTTGCATACAAAGGCAGC

Lipin2 GCCCACATAATTCATGGTTTG GGTTCAGGAAAGCTCGTTGA

ACADM AGCTCTAGACGAAGCCACGA GCGAGCAGAAATGAAACTCC

HSL CCTCCAAGCAGGGCAAAGA GCGTAAATCCATGCTGTGTGA

ATGL/PNPLA2 CCACTCACATCTACGGAGCC TAATGTTGGCACCTGCTTCA

AADAC ACCGCTTCCAGATGCTATTG TGATTCCCAAAAGTTCACCA

MTTP CGTGGTGAAAGGGCTTATTC TCGCGATACCACAGAATGAA

DGAT1 GACGGCTACTGGGATCTGA TCACCACACACCAATTCAGG

DGAT2 CGCAGCGAAAACAAGAATAA GAAGATGTCTTGGAGGGCTG

LPL TTTGGCTCCAGAGTTTGACC TGTGTCTTCAGGGGTCCTTAG

CES3/TGH TGGTATTTGGTGTCCCATCA GCTTGGGCGATACTCAAACT

CD36 GCGACATGATTAATGGCACA CCTGCAAATGTCAGAGGAAA

NR4A ATTGAGCTTGAATACAGGGCA GCTAGAAGGACTGCGGAGC

PCSK9 GGCTGCCAGGAACCTACATT CTGGGCGAAGACAAAGGAGT

LDLr GGGAACATTTCGGGGTCTGT AGTCTTCTGCTGCAACTCCG

*β*-actin TATTGGCAACGAGCGGTTCC GGCATAGAGGTCTTTACGGATGTC
