# Supplementary material for: Role of PPARα and HNF4α in Stress-Mediated Alterations in Lipid Homeostasis
Source: PLoS One. 2013 Aug 14;8(8):e70675. doi: 10.1371/journal.pone.0070675 (PMC3743822; doi:10.1371/journal.pone.0070675)
Supplement: Table S2 — Alterations in the body weight after stress. Body weight values are expressed in g. (DOC) [file pone.0070675.s002.doc]

**Table S2**

Alterations in the body weight after stress.

| Treatment | 1^st^ day | 4^th^ day |
| --- | --- | --- |
| CONTROL | 25.1±1.0 | 26.3±0.8 |
| STRESS | 25.7±0.7 | 26.7±0.6 |
