# Supplementary figures and images for: Role of PPARα and HNF4α in Stress-Mediated Alterations in Lipid Homeostasis
Source: PLoS One. 2013 Aug 14;8(8):e70675. doi: 10.1371/journal.pone.0070675 (PMC3743822; doi:10.1371/journal.pone.0070675)

**Figure S1**

**
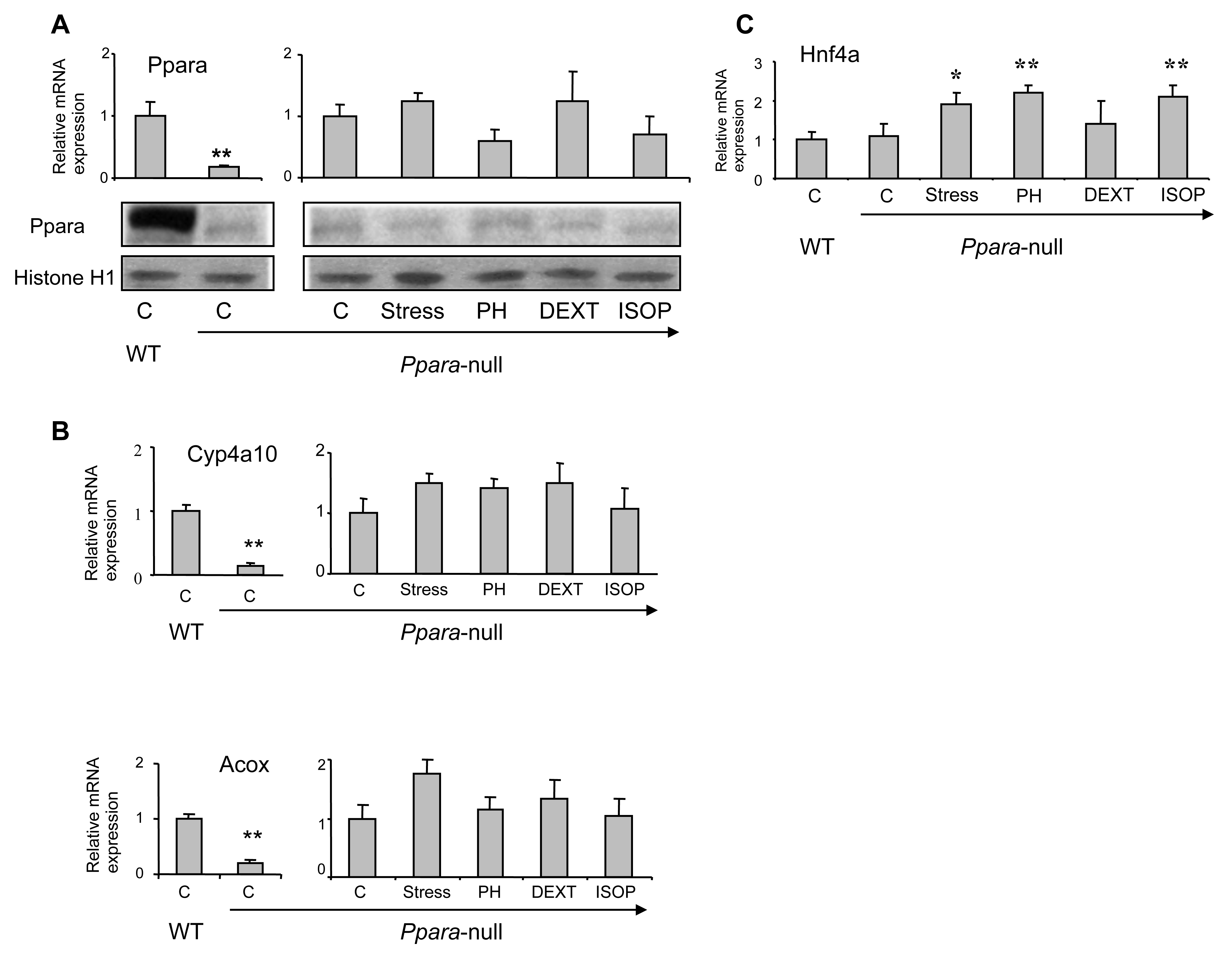
**

Supplement: Figure S1 — Stress-induced effect on PPARα expression in Pparα null mice. A. PPARα mRNA and protein levels were examined in the liver of Pparα null mice followed restraint stress or treatment with AR agonists. B. Hepatic Acox and Cyp4a10 mRNA levels were also analyzed by qPCR in these mice. C. HNF4α mRNA levels were examined in the liver of Ppara-null mice followed restraint stress or treatment with AR agonists. C: controls, PH: phenylephrine (α1-agonist), DEXT: dexmedetomidine (α2-agonist), ISOP: isoprenaline (β-agonist). Values were quantified using the comparative CT methods normalized to β-actin and are expressed as mean ± SE (n = 8–10). Comparisons took place between controls and stress-exposed or drug-treated mice. Group differences were calculated by one-way ANOVA, followed by Bonferonni's test. * P<0.025, **P<0.01. (DOC) [file pone.0070675.s003.doc]

**Figure S2**


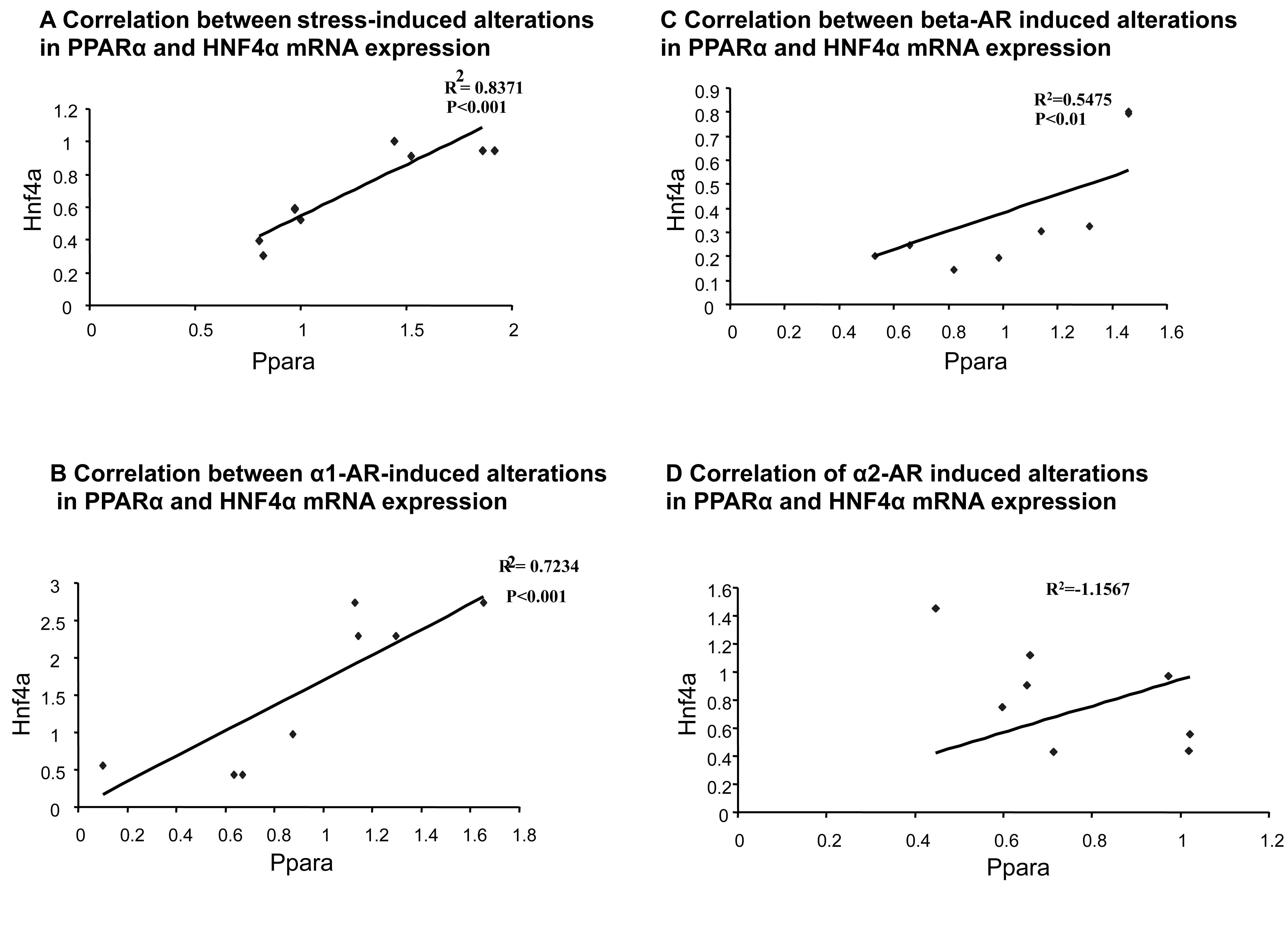

Supplement: Figure S2 — Correlation between stress- and AR-induced alterations in PPARα and HNF4α expression. A. Correlation between stress-induced alterations in PPARα and HNF4α relative mRNA expression. B. Correlation between α1-AR-induced alterations in PPARα and HNF4α relative mRNA expression. C. Correlation between β-AR induced alterations in PPARα and HNF4α relative mRNA expression. D. Correlation of α2-AR induced alterations in PPARα and HNF4α mRNA relative expression. (DOC) [file pone.0070675.s004.doc]
